# The Synergistic Activity and Optimizing Doses of Tigecycline in Combination with Aminoglycosides against Clinical Carbapenem-Resistant *Klebsiella pneumoniae* Isolates

**DOI:** 10.3390/antibiotics10060736

**Published:** 2021-06-17

**Authors:** Parnrada Nulsopapon, Worapong Nasomsong, Manat Pongchaidecha, Dhitiwat Changpradub, Piraporn Juntanawiwat, Wichai Santimaleeworagun

**Affiliations:** 1 Department of Pharmacy, Faculty of Pharmacy, Silpakorn University, Nakhon Pathom 73000, Thailand; aom.olivegreen1812@gmail.com (P.N.); pongchaidecha_m@su.ac.th (M.P.); 2 Pharmaceutical Initiative for Resistant Bacteria and Infectious Diseases Working Group [PIRBIG], Faculty of Pharmacy, Silpakorn University, Nakhon Pathom 73000, Thailand; 3 Department of Internal Medicine, Division of Infectious Diseases, Phramongkutklao Hospital and College of Medicine, Bangkok 10400, Thailand; nasomsong.w@gmail.com (W.N.); dhitiwat@yahoo.com (D.C.); 4 Department of Clinical Pathology, Division of Microbiology, Phramongkutklao Hospital, Bangkok 10400, Thailand; mamicroma@gmail.com

**Keywords:** CRE, colistin resistance, NDM, OXA-48

## Abstract

Carbapenem-resistant Enterobacteriaceae (CRE), especially carbapenem-resistant *Klebsiella pneumoniae* (CRKP), are among the largest pathogenic threats to humans. The available antibiotic treatment options for combating CRKP are limited. Colistin-resistant Enterobacteriaceae (CoRE) have also been reported worldwide, including in Thailand. Therefore, this study aimed (1) to determine minimum inhibitory concentrations (MICs) and synergistic activities of antibiotics of CRKP, and (2) to determine the probability target of attainment (PTA) and cumulative fraction of response (CFR) using pharmacokinetic/pharmacodynamic (PK/PD) data. Clinical CRKP isolates were obtained from Phramongkutklao Hospital (June to November 2020). Broth microdilution and checkerboard techniques were used to determine the mono- and synergistic activities of antibiotics. Carbapenemase and *mcr-1* genes were also identified by polymerase chain reaction (PCR). The optimal antibiotic regimens were evaluated using Monte Carlo simulations. Forty-nine CRKP isolates were collected, 40 of which were CoRKP strains. The MIC50 and MIC90 of tigecycline, amikacin, and gentamicin were 1 and 2 µg/mL, 4 and 16 µg/mL, and 0.25 and 4 µg/mL, respectively. None of any isolates expressed the *mcr-1* gene, whereas *bla_OXA-48_* (53.1%) and *bla_OXA-48_* plus *bla_NDM_* (42.9%) were detected. Synergistic activity was observed in 8.2% of isolates for tigecycline combined with amikacin or gentamicin. Additive activity was observed in 75.5% of isolates for tigecycline-amikacin and 69.4% for tigecycline-gentamicin, and no antagonism was observed. High-dose antibiotic regimens achieved the PTA target. The general recommended dose of combination regimens began with 200 mg tigecycline and 25 mg/kg amikacin, or 7 mg/kg gentamicin, followed by 100 mg tigecycline every 12 h and 15 mg/kg amikacin or 5 mg/kg gentamicin every 24 h. In conclusion, tigecycline plus aminoglycosides might be a potential regimen against CRKP and CoRKP. The appropriate combination regimen based on MIC-based dose adjustment can improve optimal antibiotic dosing. Further research via clinical studies will be necessary to confirm these results.

## 1. Introduction

Carbapenem-resistant Enterobacteriaceae (CRE), especially carbapenem-resistant *Klebsiella pneumoniae* (CRKP), is among the most present and dangerous pathogens prioritized by the World Health Organization (WHO) [1]. CRE mainly causes a variety of nosocomial infections, such as bloodstream infections, hospital acquired pneumonia (HAP), ventilator-associated pneumonia (VAP), urinary tract infections, intra-abdominal infections, and skin and soft tissue infections [1,2]. Additionally, the emergence and spread of CRE leads to significant global health issues that cause high mortality and medical expenditure [1,3]. Based on data from the Centers for Disease Control and Prevention (CDC), the death rate among patients infected with CRE bloodstream infections can be up to 50% [4]. Moreover, patients infected with CRE had higher mortality rates than those infected with carbapenem-susceptible Enterobacteriaceae (CSE) [5]. The prevalence of CRE infection has continually trended upwards over the last decade throughout the USA, Europe, and Asia [6,7,8]. In Thailand, the National Antimicrobial Resistance Surveillance Thailand (NARST) reported that the prevalence of CRKP has risen from 1.1% to 13.2% from 2010 to 2020 [9].

Currently, the available antibiotic options are limited for combating CRE. Even the efficacy of the newer beta-lactam/beta-lactamase inhibitors such as ceftazidime/avibactam, meropenem/vaborbactam, and imipenem/relebactam relies on the types of carbapenemases produced by CRE isolates, which is variable in each region. Additionally, treatment of CRE infection with combination therapy is the preferred option, as opposed to monotherapy [10,11]. Combination therapy also improves outcomes by maximizing bacterial killing and preventing bacterial regrowth [12,13].

The current combination regimen consists of mainstay antibiotics, which may be ineffective against CRE isolates, whereas other antibiotics might be more efficacious [12]. The antibiotic regimens may be divided into carbapenem-based regimens (when meropenem MICs ≤ 8 µg/mL), colistin-based regimens, and tigecycline-based regimens, whereas aminoglycosides may be often used as adjunctive antibiotics [14]. However, if the CRKP are susceptible to aminoglycosides, they are considered as part of the antibiotic regimens combined with other active antibiotics.

Tigecycline-based regimens are one option for CRE treatment, especially for intra-abdominal and skin/soft tissue infections with low risk of nephrotoxicity [2,11,15]. Currently, colistin-resistant Enterobacteriaceae (CoRE) have been reported in various regions worldwide, including Thailand [16,17,18]. Tigecycline presents an interesting choice of antibiotic against either CRE or CoRE infections. Due to its pharmacokinetic properties, the quite high volume of distribution results in tigecycline penetrates and distributed into tissues rather than the plasma. Therefore, high doses of tigecycline may increase the plasma concentrations, leading to adequate bactericidal activity against CRE isolates [2].

Amikacin and gentamicin are most frequently selected as the first adjunctive agents in combination regimens. Many previous studies revealed the excellent activity against CRE isolates [19,20,21]. In Thailand, *K. pneumoniae* isolates have been susceptible to amikacin and gentamicin at rates of 94.7% and 82.8%, respectively [22]. The combination of tigecycline with amikacin or gentamicin is one of the most efficacious combinations in the antibiotic-resistant era of CRKP and colistin-resistant *K. pneumoniae* (CoRKP). Although the previous studies described the synergistic activities of these combinations, they have not drawn clear conclusions because the variety of study methodologies, antimicrobial susceptibility profiles, and different carbapenemase types have led to inconsistent findings [14,15]. The Monte Carlo simulation is an advanced statistical technique that randomly chooses pharmacokinetic parameters and distributions, integrated with pharmacokinetic equations and pharmacokinetic/pharmacodynamic (PK/PD) indices, to estimate and predict the optimal dosage regimens [23].

In this study, we conducted in vitro experiments with combinations of tigecycline and amikacin or gentamicin to determine antimicrobial susceptibility and the synergistic activity of these antibiotics against CRKP and CoRKP clinical isolates. We also generated an appropriate dosage regimen using PK/PD targets for the treatment of critically ill patients infected with CRKP or CoRKP.

## 2. Materials and Methods

### 2.1. Bacterial Strains

A total of 49 non-duplicate CRKP strains were collected from patients admitted to the Pharmongkutklao Hospital, Bangkok, Thailand, during the period from June to November 2020. Various clinical specimens were obtained from different individual patients infected with CRKP. All studied strains were identified as *K. pneumoniae* by using Matrix-Assisted Laser Desorption/Ionization Time-of-Flight Mass Spectrometry (MALDI-TOF MS). After that, antimicrobial susceptibility testing was performed using the broth microdilution method (Sensititre, TREK Diagnostic Systems, Inc., Cleveland, OH, USA) and interpreted following Clinical and Laboratory Standards Institute (CLSI) 2020 for identification as CRKP [24].

After getting samples, all strains were isolated again on sheep blood agar (Clinical Diagnostics Ltd., Part, Bangkok, Thailand) and purified on MacConkey agar (Clinical Diagnostics Ltd., Part, Bangkok, Thailand). To identify *K. pneumoniae,* the morphology of colonies on MacConkey agar appeared large and mucoid-pink. Furthermore, meropenem minimum inhibitory concentrations (MICs) were determined by broth microdilution to confirm CRKP strains.

CRKP isolates were defined as non-susceptible to either ertapenem, imipenem, or meropenem according to the procedures described by the Clinical and Laboratory Standards Institute (CLSI) 2020 [24]. All CRKP strains were stored at −80 °C until analysis. *Escherichia coli (E. coli)* ATCC 25922 was used as a reference strain for quality control.

### 2.2. Determining MICs and Fractional Inhibitory Concentration Indices (FICI)

#### 2.2.1. Determining MICs

MIC values were determined using broth microdilution. Two-fold serial dilutions of colistin, tigecycline, amikacin, and gentamicin ranged from 0.0625 to 128 µg/mL, 0.03125 to 128 µg/mL, 0.0625 to 128 µg/mL, and 0.03125 to 64 µg/mL, respectively. Furthermore, a purified single colony of isolated strains was picked up and suspended to 0.9% in normal saline (Univar^®^, Ajax Finechem Pty Ltd, Taren Point, Australia). Next, the bacterial suspension was adjusted to a turbidity equivalent to 0.5 McFarland standard. After that, the prepared bacterial suspension (~10^8^ cfu/mL) was diluted in cation adjusted Mueller Hinton Broth (BBL™ Mueller Hinton II Broth (Cation-Adjusted), Becton Dickinson and company, France) at 1:1000 dilution. The final bacterial suspension contained an inoculum density of ~10^5^ cfu/mL. Then, 50 µL of two-fold serial antibiotic dilutions and 50 µL of the final inoculum were dispensed into 96-well microplates. MICs were determined after incubation at 37 °C for 18–20 h [24]. MIC breakpoints were interpreted according to the European Committee on Antimicrobial Susceptibility Testing breakpoint for 2020 (EUCAST, 2020) for tigecycline and fosfomycin [25], as was the CLSI breakpoint in 2020 for other antibiotics [24]. The cut-off MIC intermediate breakpoint was ≤2 µg/mL for colistin. In addition, the cut-off MIC susceptible breakpoints were ≤0.5 µg/mL for tigecycline, ≤16 µg/mL for amikacin, ≤4 µg/mL for gentamicin, ≤32 µg/mL for fosfomycin, ≤8 or 4 µg/mL for ceftazidime/avibactam, ≤1 µg/mL for meropenem and for imipenem, and ≤4 µg/mL for aztreonam.

MIC50 is defined as the MIC value at which ≥50% of the isolates in a test population are inhibited. MIC90 is defined as the MIC value at which ≥90% of the isolates in a test population are inhibited.

#### 2.2.2. Performing FICI

The synergistic effect of antibiotic combinations including tigecycline-amikacin and tigecycline-gentamicin was determined using the checkerboard technique. The studied antibiotic concentrations of a series of two-fold dilutions were 0.125–8 µg/mL for tigecycline, 0.0625–64 µg/mL for amikacin, and 0.0078–8 µg/mL for gentamicin.

Of the checkerboard, the MIC of either antibiotic combination or antibiotic alone is the lowest drug concentration that contains no growth (a clear on the well) [26,27]. The FICI was defined as the aggregate MIC ratio of each combination. It was calculated as follows:

FICI = ∑FIC_AB_ = FIC_A_ + FIC_B_ (1)

where FIC_A_ = MIC of drug A in combination/MIC A alone; FIC_B_ = MIC of drug B in combination/MIC B alone.

A FICI index specified the effects of a given combination, which were classified into 4 categories. Synergism, additive effects, indifference, and antagonism were indicated as an ∑FIC index of ≤0.5, an ∑FIC index of >0.5–1, an ∑FIC index of >1–4, and an ∑FIC index of >4, respectively [28].

### 2.3. Molecular Study of Antibiotic Resistance Genes

The carbapenemase types in all studied *K. pneumoniae* strains were evaluated using the polymerase chain reaction (PCR) method. Multiplex PCRs consisted of multiple primer sets of carbapenemase genes (*bla_IMP_, bla_VIM_, bla_OXA-48_, bla_NDM_*_,_ and *bla_KPC_*); moreover, the *mcr*-1 primer also detected *mcr-1* gene isoforms. The primer pairs of each gene are presented in Table 1.

The multiplex PCR conditions for amplification were as follows: preincubation for 3 min at 94 °C, 35 cycles of 30 s at 94 °C, 35 s at 57 °C, and 45 s at 72 °C; and a final extension step for 5 min at 72 °C [31]. The *mcr*-1 PCR conditions were as follows: preincubation for 5 min at 94 °C, 35 cycles of 30 s at 94 °C, 35 s at 53 °C, and 45 s at 72 °C; and a final extension step for 5 min at 72 °C. The amplified products were analyzed by agarose (1%) gel electrophoresis in 0.5 × Tris/Borate/EDTA (TBE), after staining with ethidium bromide. The criterion for identifying the resistant genes was identical positions of bands between the PCR products and the PCR reference.

### 2.4. Antibiotic Dose Optimization by Monte Carlo Simulation

All pharmacokinetic parameters of critically ill patients were obtained from previous studies. A set of population pharmacokinetic parameters is shown in Supplementary Materials S1 (Tables S1–S3). The relationship between drug concentration levels and time was generated using a two-compartment model for tigecycline and amikacin and a one-compartment model for gentamicin with the linear pharmacokinetic behavior [32,33,34]. For amikacin and gentamicin, creatinine clearance (CrCL) was included to simulate the plasma aminoglycoside concentration-time using the equations containing CrCL as covariate factor (Supplementary Materials S1: Tables S1–S3).

The PD indies for tigecycline are characterized by the area under the curve (AUC) of the unbound tigecycline plasma concentration-time at 24 h to the MIC, whereas amikacin and gentamicin used the C_max_/MIC ratio. The effective PK/PD targets and indices of tigecycline and aminoglycosides were *f*AUC_0–24_/MIC ≥ 0.9 and C_max_/MIC > 8, indicative of good clinical outcomes [35,36]. The safe PK/PD targets of aminoglycosides were AUC_0–24_ (mg × h/L) > 700 which occurred nephrotoxicity rate more than 10% [37]. The trapezoidal rule was used to calculate AUC. *f*AUC was calculated and divided by the given MIC to estimate the desired PK/PD value; the tigecycline unbound fraction of 80% was used.

The regimens of tigecycline ranged from a loading dose of 200–400 mg, followed by 100–200 mg intravenous injection every 12–24 h in general patients, whereas the regimens of amikacin and gentamicin ranged from a loading dose of 15–30 mg/kg, followed by 7.5–20 mg/kg and a loading dose of 3–8 mg/kg, followed by 2.5–7 mg/kg every 24–48 h, respectively. Different PK profiles were simulated only for aminoglycosides with patients having creatinine clearance of <10 mL/min, 10–25 mL/min, 26–50 mL/min, and 51–90 mL/min, respectively. The dosing regimens were simulated over a 24 h period. Details of the antibiotic dosing regimen are shown in Supplementary Materials S1 (Table S4).

The optimal antibiotic regimens were investigated using a 10,000-subject Monte Carlo simulation (Oracle Crystal Ball Classroom Faculty Edition-Oracle 1-Click Crystal Ball 201, Thailand).

MCS is a mathematical technique to simulate 10,000 virtual patients based on mean pharmacokinetic parameters (drug clearance or volume of distribution) and their distribution (standard derivation) in the compartment models (one or two compartments) for computing the plasma concentration and time among individual virtual patients. The Cmax and calculated AUC values from the trapezoidal rule among simulated patients and the probability of target attainment (PTA) of the target PK/PD index were determined.

The PTA was defined as the predicted probability that each antibiotic regimen would meet a PK-PD index and a PK-PD target at each MIC. Additionally, the cumulative fraction of response (CFR) was defined as the sum of the cumulative fraction of each regimen, calculated as the fraction of bacteria multiplied by the PTA of each antibiotic MIC. The optimal dosing regimens defined as the dosing regimens of each antibiotic MIC or the MIC values from the synergistic study reached greater than 90% of PTA for documented therapy and greater than 90% of CFR for empirical therapy. Each dosing regimen for tigecycline, amikacin, and gentamicin, we estimated the PTA to achieve MIC ranging from 0.0625 to 16 μg/mL in the target. CFR calculation was achieved using the MIC distributions.

### 2.5. Ethics Approval

Ethical approval for this study was obtained from the ethics review committee of the Royal Thai Army Medical Department and Phramongkutklao Hospital, Bangkok, Thailand (Ethics number: Q011h/63 issued on 13 July 2020).

## 3. Results

### 3.1. In Vitro Susceptibility of Tigecycline, Amikacin, and Gentamicin

Overall results of the antibiotic-susceptible and the antibiotic-resistant genes for all CRKP isolates are presented in Table 2. Forty-nine CRE isolates were collected from various human patients. Among the studied antibiotics, the CRKP isolates were susceptible to amikacin (91.8%), gentamicin (89.8%), and ceftazidime/avibactam (55.1%), whereas they were resistant to meropenem (100%), aztreonam (98%), fosfomycin (92%), imipenem (88%), colistin (82%), and tigecycline (79.6%). The MIC50, MIC90, MIC range to tigecycline, amikacin, and gentamicin are reported in Table 2.

No isolates were found to express the *mcr-1* gene, *bla_IMP_*, *bla_VIM_,* and *bla_KPC_*. In the total of 49 isolates, most isolates expressed the carbapenemase encoded by the *bla_OXA-48_* gene (26 of 49; 53.1%), followed by isolates harboring both *bla_OXA-48_* plus *bla_NDM_* (21 of 49, 42.9%) and *bla_NDM_* (1 of 49, 2%), respectively. One strain was not found to contain any carbapenemase.

### 3.2. Synergistic Activities

Synergy was observed in the same percentages of isolates (4 of 49; 8.2%). Additive activity was mostly shown in 37 of 49 (75.5%) isolates for tigecycline-amikacin and 34 of 49 (69.4%) for tigecycline-gentamicin, whereas indifferent activities of tigecycline-gentamicin and tigecycline-amikacin were observed in 11 (22.4%) and 8 (16.3%) of 49 isolates, respectively. Antagonism was not observed in any checkerboard assays.

Interestingly, both antibiotic combinations had synergistic or additive activities against 40 CoRKP isolates. Synergy was found for 3 (7.5%) and 4 (10.0%) of 40 for tigecycline plus amikacin and tigecycline plus gentamicin, respectively. Whereas, additive activities were shown in 29 isolates (72.5%) for tigecycline plus amikacin and 34 (62.5%) of 40 isolates for tigecycline plus gentamicin, respectively. Focusing on carbapenemase genes, tigecycline plus amikacin had synergistic activity against OXA-48-like producing isolates (3 of 40; 7.5%), whereas tigecycline plus gentamicin had these activities against either OXA-48-like producing (3 of 40; 7.5%) or OXA-48-like plus NDM-producing (1 of 40; 2.5%) isolates. The results of the checkerboard synergy testing are shown in Table 2 and Table 3. Details of the MIC distribution of single or combined antibiotics are shown in Supplementary Materials S2.

### 3.3. PTA and CFR

For tigecycline dosage regimens to meet the PTA at *f*AUC_0–24_/MIC ≥ 0.9, once daily high-dose tigecycline (400 mg loading dose) was followed by 200 mg supplemental doses every 12 h, which achieved ≥90% PTA with a MIC of 2 µg/mL. Twice daily tigecycline doses achieved the PTA target with a MIC of 1 µg/mL, whereas all tigecycline dosage regimens reached the PTA target of a MIC of 0.5 µg/mL. The assessments of PTA and CFR for different tigecycline dosages are shown in Table 4.

The aminoglycoside dosage regimens also reached the PTA target at C_max/MIC_ > 8. At a MIC of 1 µg/mL, all amikacin dosage regimens met the PTA target in critically ill patients. At a MIC of 2 µg/mL, a loading dose of 25–30 mg/kg followed by a maintenance dose of 15–20 mg/kg of amikacin met the PTA target in patients with creatinine clearance levels of more than 50 mL/min. No regimens achieved the target at MIC50 and MIC90. For gentamicin, all dosage regimens met the PTA target in critically ill patients with an MIC of 0.25 (MIC50) and 0.5 µg/mL. At a MIC of 1 µg/mL, 4 mg/kg and 5 mg/kg once daily regimens (extended infusion) reached the PTA target in patients with creatinine clearance at 10–50 and 51–90 mL/min, respectively. No regimens achieved the target with a gentamicin MIC90 of 4 µg/mL, nor did any regimen of aminoglycosides achieve the CFR target when used as single agents, whereas all regimens of aminoglycosides met the target when combined with other antibiotic agents. The PTA and CFR values for different aminoglycoside regimens are evaluated in Table 5 and Table 6.

## 4. Discussion

In these studies, we determined the microbial susceptibilities of CRKP isolates to tigecycline and aminoglycosides. In our findings (Table 2), one-fifth (20.4%) of CRKP isolates were only susceptible to tigecycline. Nonetheless, previous studies reported tigecycline susceptibility rates ranged from 12 to 100% [18,21,38,39] in Thailand, as well as 58.0–86.1% in other countries [19,40]. It is possible that there are differences in the cutoff tigecycline breakpoint (in a range of between 0.5 and 2 µg/mL). Furthermore, growing rates of CRKP and CoRKP in our setting may lead to increased use of tigecycline [9,18].

From Table 4, all high-dose tigecycline regimens reached the PTA target for isolates with an MIC of 0.5 µg/mL. At MIC = 1 µg/mL, a loading dose of 200 mg of tigecycline followed by a maintenance dose of 100 mg every 12 h (400 mg/day) was able to reach the PTA target; these results were consistent with those of previous studies [41,42]. At MIC = 2 µg/mL, our results showed that a loading dose of 400 mg tigecycline followed by a maintenance dose of 200 mg every 12 h (400 mg/day) also achieved the PTA targets. Based on tigecycline PK/PD, once daily high doses of tigecycline (loading dose of 400 mg followed by maintenance doses of 200 mg every 24 h) also met the PTA target with an MIC of 1 µg/mL. Thus, high-dose tigecycline regimens with once daily maintenance doses should be considered for the treatment of infections caused by CRKP or CoRKP, as these antibiotics have a concentration-dependent killing property and long half-life (~27–42 h) [43].

Most CRKP isolates were found to be susceptible to aminoglycosides (approximately 90%). These findings from Table 2 were consistent with previous findings from NARST. The susceptibility rates to amikacin and gentamicin ranged between 80% and 100% [18,21,22]. Furthermore, we observed that all isolates were only resistant to one of the two aminoglycosides tested. The occurrence of aminoglycoside-modifying enzymes (AMEs) may be one mechanism of resistance, being associated with no cross-resistance to all aminoglycosides [44]. High-dose regimens of aminoglycosides were simulated for critically ill patients in order to increase the probability of achieving target peak concentrations. Our findings (Table 5 and Table 6) showed the regimens of amikacin and gentamicin met the PTA targets (at C_max/_MIC = 8) with MICs of no more than 2 and 1 µg/mL, respectively. An earlier study showed aminoglycoside dosage regimens (ranged from 5 to 30 mg/kg) achieved the PTA target at MICs of 0.5 µg/mL with the corresponding target of C_max_/MIC ≥ 10 [33]. Although our study used the minimum PK/PD target of aminoglycosides when compared with the previous study, the dosage regimens did not achieve the targets within the susceptibility breakpoints. Thus, aminoglycosides should be used as the adjuncts for the treatment of infections caused by CRKP or CoRKP.

Our findings showed high colistin resistance rates of CRKP isolates (*n* = 40 of 49; 81.6%) at high concentrations (colistin MICs ≥ 16 µg/mL; *n* = 32 of 40; 80.0%) in Table 2. In prior studies, colistin resistance rates of CRKP isolates were only 25% [39], whereas colistin resistance rates of *K. pneumoniae* isolates ranged from 17.3% to 76.1% [18,45,46,47]. Not surprisingly, high rates of colistin resistance were reported because our study only collected *K. pneumoniae* isolates, which had the highest rates of colistin resistance among Gram-negative bacteria [18].

Generally, colistin-based regimens were one of the active regimens for treatment if the infections were found to be susceptible to colistin [14,48]. Our results found that 80% of CRKP isolates had high MICs of colistin (MICs ≥ 16 µg/mL). A higher colistin MIC could lead to treatment failure and development of antibiotic resistance [49]. Furthermore, a PK/PD study recommended colistin MICs to achieve probability of target attainments (PTAs) in all patients with various creatinine clearances ranging from 0.5 to 16 µg/mL [46]. Hence, colistin combination regimens may not be recommended to treat patients in our setting.

Tigecycline-based regimens are alternative regimens for the treatment of multi-drug resistant bacteria with high MIC values. The results of checkerboard assays from Table 2 showed the sum of in vitro interaction of the combination of tigecycline with amikacin and gentamicin. These assays generated a potent effect greater than or equal to that of the individual antibiotics. Overall, four isolates showed synergism with both combinations, whereas the additive effects of tigecycline plus gentamicin and tigecycline plus amikacin were seen in 34 and 37 isolates, respectively (Table 3). Related studies also reported tigecycline plus amikacin or gentamicin was effective against *K. pneumoniae* isolates. For tigecycline plus amikacin, one study showed the synergistic effect was 36.4%, whereas another study found the synergistic effect was 70% [19,50]. For tigecycline plus gentamicin, one study showed a synergistic increase of 28.6%, whereas another study showed only 13.3% [19,21]. Notably, our results were not consistent with previous studies, likely because of the differences in tigecycline susceptibility and the type of multi-drug resistance genes present.

The synergistic activities of these antibiotics may be associated with co-resistance genes. Our study showed 8.2% synergy rates, whereas a previous study (Prawang et al.) collected the isolates in the same hospital during 2016 to 2018 and showed 13.3% synergy rates [21]. Although we conducted a study in the same setting, a difference in the percentage of synergy was found. The different results may be caused by the genetic background. Our isolates contained co-resistance of carbapenemase gene (*bla_OXA-48-like_* (53.1%) and *bla_OXA-48-like_* plus *bla_NDM_* (42.9%), whereas the isolates of a previous study contained only *bla_OXA-48-like_* (~90%). Notably, the CRKP isolates producing OXA-48-like carbapenemase showed more antibiotic synergy than the CRKP isolates producing NDM carbapenemase. Coproduction of NDM-1 and aminoglycoside resistance genes (e.g., 16S Ribosomal RNA Methyltransferases F: RmtF) may be associated with mobile genetic elements in CRKP isolates, leading to a high level of resistance to aminoglycosides [51]. Recent studies also showed synergy of tigecycline and aminoglycosides in KPC-2-producing CRKP isolates ranging from 36.4% to 70% [19,50]. Therefore, these combinations may be used as a first choice of treatment in many regions and countries, based on epidemic spreading characteristics.

The combinations of these drugs have an activity against CRKP isolates. When two antibiotics were taken to the bacteria cells, the synergism may occur because of two reasons. Firstly, tigecycline binds to the bacterial ribosome and inhibits protein synthesis. Secondly, aminoglycosides may disrupt and increase permeability of the bacterial outer membrane [52,53]. In a comparison of two aminoglycosides, amikacin can increase the permeability of cell membrane more than gentamicin [54]. Aminoglycosides lead to mistranslated amino acids, in turn leading to damage to the bacterial cytoplasmic membrane and interfering with protein synthesis; these protein errors also lead to reduced β-lactamase expression from aminoglycoside activities. Therefore, aminoglycosides likely enhance combined antibiotics to the target sites, contributing to synergistic effects [52,53]. Moreover, the combination of antibiotics could also decrease mutational frequencies by a range of 1000–10,000-fold at low concentration (1× MIC), suppressing the emergence of resistance genes [55].

The synergistic or additive effects lead to a reduction in MICs. Our findings from Table 2 showed MIC50, MIC90, and maximum MICs of tigecycline, amikacin, and gentamicin in combination regimens decreased by 1- to 4-fold when compared to single antibiotic regimens. Consequently, the MICs moved from resistant to susceptible, leading to achievement of PTA and CFR targets with low-dose regimens [56]. These beneficial impacts are associated with low rates of adverse drug reactions, particularly nephrotoxicity and ototoxicity from aminoglycosides, and lead to high rates of successful treatment [10,11,48]. It is reasonable to suggest the combination of tigecycline with amikacin or gentamicin for the treatment of severe infections caused by CRE, especially in the polymyxin-resistance era.

This study has several limitations. Firstly, our study might not be generalizable to other settings because the collection was made from a single university hospital. Secondly, our results were obtained from in vitro experiments using stable concentrations of antibiotics, and the therapeutic efficacy from patients’ immune response was not taken into account. Third, we did not perform other mechanisms (e.g., efflux pumps and porin loss). Whole-genome sequencing should be investigated to obtain further information on the genetic variants associated with antibiotic resistance genes. Lastly, tigecycline with aminoglycosides demonstrated higher activities in vitro and may be useful for treatment of infections caused by CRE in hospitals. Further studies in clinical patient-related outcomes are required to confirm their clinical use.

## 5. Conclusions

Tigecycline plus amikacin or gentamicin showed synergistic or additive effects against CRKP and CoRKP isolates. The benefit of such combinations might improve the PTA and CFR achievement, and also reduce the risk of nephrotoxicity due to a lower dose of aminoglycosides. A large sample of isolates in vitro and from clinical patient-related outcomes should be assessed to confirm the combinatory activities of these antibiotics against CRKP and CoRKP isolates.

## Figures and Tables

**Table 1:** Primer sets for detection genes of antibiotic resistance [29,30].

Type of Resistance Genes	Primers
*IMP*	IMP-F: 5′-GGAATAGAGTGGCTTAAYTCTC-3′ IMP-R: 5′-GGTTTAAYAAAACAACCACC-3′
*VIM*	VIM-F: 5′-GATGGTGTTTGGTCGCATA-3′ VIM-R: 5′-CGAATGCGCAGCACCAG-3′
*OXA-48*	OXA 48-F: 5′-GCGTGGTTAAGGATGAACAC-3′ OXA 48-R: 5′-CATCAAGTTCAACCCAACCG-3′
*NDM*	NDM-F: 5′-GGTTTGGCGATCTGGTTTTC-3′ NDM-R: 5′-CGGAATGGCTCATCACGATC-3′
*KPC*	KPC-F: 5′-CGTCTAGTTCTGCTGTCTTG-3′ KPC-R: 5′-CTTGTCATCCTTGTTAGGCG-3′
*mcr-1*	MCR-1-F: 5′-CGG TCA GTC CGT TTG TTC-3′ MCR-1-R: 5′-CTT GGT CGG TCT GTA GGG-3′

**Table 2:** Results of antibiotic susceptibility from the broth microdilution method, the antibiotic resistant genes from multiplex polymerase chain reaction (MTP-PCR), and synergy from the checkerboard method for all carbapenem-resistant *Klebsiella pneumoniae* (CRKP) isolates (*n* = 49) divided by colistin breakpoints according to Clinical and Laboratory Standards Institute (CLSI) guidelines.

No	Specimens	MTP PCR	MICs (µg/mL)			Synergistic Testing			
			AMK	GEN	TGC	AMK + TGC		GEN + TGC	
						∑ FICI	Interpretation	∑ FICI	Interpretation
Colistin-intermediate strains									
1	Blood	OXA	2	≤0.03125	2	≤0.625	ADD	≤0.5625	ADD
2	Blood	OXA	8	≤0.03125	2	≤0.5625	ADD	≤0.5625	ADD
3	Blood	OXA	8	≤0.03125	2	≤0.5313	ADD	0.75	ADD
4	Sputum	OXA	0.25	≤0.03125	1	≤0.75	ADD	0.625	ADD
5	Sputum	OXA	8	≤0.03125	2	≤0.375	SYN	≤0.5313	ADD
6	Urine	OXA	8	0.0625	2	1	ADD	0.5625	ADD
7	Urine	OXA	2	0.25	2	≤0.5625	ADD	≤0.5313	ADD
8	Urine	OXA	2	≤0.03125	1	0.75	ADD	0.625	ADD
9	Urine	OXA	2	0.25	2	≤0.5625	ADD	≤0.5313	ADD
Colistin-resistant strains									
10	Blood	OXA + NDM	8	8	1	≤0.75	ADD	≤1.002	IND
11	Blood	OXA + NDM	8	8	1	2	IND	≤1.0039	IND
12	Blood	OXA + NDM	8	16	4	≤0.75	ADD	≤0.5313	ADD
13	Blood	OXA + NDM	4	8	1	≤0.75	ADD	≤1.002	IND
14	Blood	OXA	2	2	8	0.5	SYN	≤1.0625	IND
15	Blood	OXA + NDM	2	≤0.03125	2	≤0.75	ADD	≤1.0625	IND
16	Blood	OXA	0.5	0.125	0.5	0.75	ADD	≤0.5313	ADD
17	Blood	OXA	16	64	2	0.75	ADD	0.75	ADD
18	Blood	OXA + NDM	32	0.25	2	0.75	ADD	≤1.0156	IND
19	Blood	OXA	2	0.125	2	≤0.625	ADD	≤1.0625	IND
20	Blood	OXA + NDM	4	0.0625	2	≤0.5625	ADD	0.625	ADD
21	Blood	OXA + NDM	8	≤0.03125	2	≤0.5625	ADD	0.375	SYN
22	Blood	OXA + NDM	8	0.125	1	≤0.75	ADD	0.625	ADD
23	Blood	OXA + NDM	16	0.125	1	0.75	ADD	0.75	ADD
24	Sputum	OXA + NDM	2	≤0.03125	1	≤0.75	ADD	≤1.0625	IND
25	Sputum	NDM	8	4	2	0.75	ADD	0.75	ADD
26	Urine	OXA	2	0.125	1	≤0.75	ADD	≤0.5313	ADD
27	Sputum	OXA	2	0.125	1	0.75	ADD	0.75	ADD
28	Urine	OXA	32	0.5	1	≤0.5	SYN	≤0.2656	SYN
29	Sputum	OXA + NDM	16	4	1	0.625	ADD	≤1.001	IND
30	Sputum	OXA	4	2	0.5	≤0.75	ADD	≤0.625	ADD
31	Urine	OXA + NDM	8	0.5	0.5	≤0.5313	ADD	0.625	ADD
32	Urine	OXA + NDM	4	0.25	0.5	≤1.125	IND	≤1.0313	IND
33	Sputum	OXA	2	0.125	0.5	≤1.125	IND	≤0.5625	ADD
34	Urine	OXA	2	2	1	0.375	SYN	≤0.5313	ADD
35	Sputum	OXA	4	0.25	1	≤0.5625	ADD	≤0.5313	ADD
36	Sputum	OXA	16	0.5	1	≤1.0078	IND	≤0.5156	ADD
37	Sputum	OXA + NDM	2	0.125	0.5	≤1.0625	IND	1	ADD
38	Sputum	OXA	1	0.125	0.5	0.75	ADD	0.625	ADD
39	Sputum	OXA + NDM	8	2	0.5	≤0.5156	ADD	≤1.002	IND
40	Sputum	OXA	2	2	1	≤0.625	ADD	0.5	SYN
41	Sputum	OXA	2	0.125	1	0.75	ADD	0.75	ADD
42	Penis	OXA + NDM	32	2	1	0.625	ADD	0.5313	ADD
43	Peritoneal fluid	OXA + NDM	32	2	2	≤1.0078	IND	1	ADD
44	Urine	OXA + NDM	1	0.125	0.5	≤1.125	IND	1	ADD
45	Urine	OXA	8	0.25	2	≤0.5156	ADD	≤0.5156	ADD
46	Sputum	OXA + NDM	1	0.25	0.5	0.75	ADD	0.625	ADD
47	Sputum	OXA + NDM	4	0.25	2	≤1.25	IND	0.75	ADD
48	Bronchoalveolar lavage	OXA	8	2	2	≤0.5625	ADD	0.5	SYN
49	Sputum	Not detected	2	0.5	16	≤0.625	ADD	0.5313	ADD
MIC50			4	0.25	1				
MIC90			16	8	2				
Min			0.25	≤0.031	0.5				
Max			32	64	16				

Abbreviations: OXA, oxacilinase-48; TGC, tigecycline; AMK, amikacin; GEN, gentamicin; FICI, fractional inhibitory concentration index; NDM, New Delhi metallo-beta lactamase; SYN, synergistic effect (FICI ≤ 0.5); ADD; additive effect (FICI 0.5 – ≤ 1); IND, indifference (FICI = 1–4).

**Table 3:** In vitro checkerboard synergistic testing against 49 CRKP isolates (*n* = 49) based on carbapenemase genes.

Antibiotic Combination	Synergism	Additive Effect	Indifference
	No (%)	No (%)	No (%)
**Tigecycline + Amikacin**			
OXA + NDM	-	15	6
OXA	4	20	2
NDM	-	1	-
Not detect of studied genes	-	1	-
Total	4 (8.2)	37 (75.5)	8 (16.3)
**Tigecycline + Gentamicin**			
OXA + NDM	1	11	9
OXA	3	21	2
NDM	-	1	-
Not detect of studied genes	-	1	-
Total	4 (8.2)	34 (69.4)	11 (22.4)

Abbreviations: OXA, oxacilinase-48; NDM, New Delhi metallo-beta lactamase

**Table 4:** The probability of target attainment (PTA) for the different tigecycline regimens in critically ill patients at steady state with targets of *f*AUC24/MIC ≥ 0.9.

Dosage Regimens		PTA (%)								
		TGC MIC (µg/mL)								
LD	MD	0.0625	0.125	0.25	0.5	1	2	4	8	16
200 mg	100 mg q 12 h	100	100	100	100	100	53	0	0	0
200 mg	100 mg q 24 h	100	100	100	100	55	0	0	0	0
400 mg	100 mg q 12 h	100	100	100	100	100	55	0	0	0
400 mg	100 mg q 24 h	100	100	100	99.99	55	0	0	0	0
400 mg	200 mg q 12 h	100	100	100	100	100	100	54	0	0
400 mg	200 mg q 24 h	100	100	100	100	100	55	0	0	0

Abbreviations: AUC, area under the curve; MIC, minimum inhibitory concentration; LD, loading dose; MD, maintenance dose; TGC, tigecycline.

**Table 5:** The PTA and cumulative fraction of response (CFR) for the different amikacin regimens in critically ill patients according to kidney function (creatinine clearance) at steady state with targets of C_max_/MIC > 8 (for efficacy) and AUC_0–24_ > 700.

Dosage Regimens		PTA (%)										CFR (%)	
		AMK MIC (µg/mL)									AUC_0–24_ > 700	AMK (mono)	AMK (with TGC)
LD	MD	0.0625	0.125	0.25	0.5	1	2	4	8	16			
Creatinine clearance 0–9 mL/min													
15 mg/kg	7.5 mg/kg q 48 h	100	100	100	100	100	67	0	0	0	11.1	32.1	95.9
20 mg/kg	7.5 mg/kg q 48 h	100	100	100	100	100	70	0	0	0	13.4	32.9	96.3
25 mg/kg	7.5 mg/kg q 48 h	100	100	100	100	100	70	0	0	0	15.4	33.2	96.4
Creatinine clearance 10–25 mL/min													
20 mg/kg	10 mg/kg q 48 h	100	100	100	100	100	89	0	0	0	24.9	39.3	98.6
20 mg/kg	15 mg/kg q 48 h	100	100	100	100	100	99	5	0	0	53.1	43.2	99.9
25 mg/kg	10 mg/kg q 48 h	100	100	100	100	100	90	0	0	0	26.8	39.6	98.8
25 mg/kg	15 mg/kg q 48 h	100	100	100	100	100	99	5	0	0	55.7	43.3	99.9
Creatinine clearance 26–50 mL/min													
20 mg/kg	12 mg/kg q 24 h	100	100	100	100	100	99	7	0	0	84.2	43.5	99.9
20 mg/kg	15 mg/kg q 24 h	100	100	100	100	100	100	19	0	0	92.7	45.2	100
25 mg/kg	12 mg/kg q 24 h	100	100	100	100	100	100	7	0	0	84.2	43.6	99.9
25 mg/kg	15 mg/kg q 24 h	100	100	100	100	100	100	21	0	0	92.7	45.4	100
Creatinine clearance 51–90 mL/min													
25 mg/kg	15 mg/kg q 24 h	100	100	100	100	100	100	18	0	0	91.9	45.1	100
25 mg/kg	20 mg/kg q 24 h	100	100	100	100	100	100	46	0	0	97.6	48.5	100
30 mg/kg	15 mg/kg q 24 h	100	100	100	100	100	100	18	0	0	91.7	45.1	100
30 mg/kg	20 mg/kg q 24 h	100	100	100	100	100	100	47	0	0	97.8	48.7	100
Creatinine clearance 91–130 mL/min													
25 mg/kg	15 mg/kg q 24 h	100	100	100	100	100	100	22	0	0	91.3	46	100
25 mg/kg	20 mg/kg q 24 h	100	100	100	100	100	100	53	0	0	97.9	49	100
30 mg/kg	15 mg/kg q 24 h	100	100	100	100	100	100	23	0	0	91.9	46	100
30 mg/kg	20 mg/kg q 24 h	100	100	100	100	100	100	52	0	0	97.7	49	100

Abbreviations: AUC, area under the curve; MIC, minimum inhibitory concentration; LD, loading dose; MD, maintenance dose; TGC, tigecycline; AMK, amikacin.

**Table 6:** The PTA for the different gentamicin regimens in critically ill patients according to kidney function (creatinine clearance) at steady state with targets of C_max_/MIC > 8 (for efficacy) and AUC_0–24_ > 700.

Dosage Regimens		PTA (%)										CFR (%)	
		GEN MIC (µg/mL)									AUC_0–24_ > 700	GEN (mono)	GEN (with TGC)
LD	MD	0.0625	0.125	0.25	0.5	1	2	4	8	16			
Creatinine clearance 0–9 mL/min													
3 mg/kg	2.5 mg/kg q 48 h	100	100	100	100	99	42	0	0	0	42.7	76.2	100
5 mg/kg	2.5 mg/kg q 48 h	100	100	100	100	99	41	0	0	0	43.3	76.0	100
7 mg/kg	2.5 mg/kg q 48 h	100	100	100	100	99	42	0	0	0	43.8	76.2	100
Creatinine clearance 10–25 mL/min													
5 mg/kg	4 mg/kg q 48 h	100	100	100	100	91	8	0	0	0	6.4	70.7	100
7 mg/kg	3 mg/kg q 48 h	100	100	100	100	78	3	0	0	0	2.4	69.8	100
7 mg/kg	5 mg/kg q 48 h	100	100	100	100	97	17	0	0	0	13.0	72.0	100
8 mg/kg	3 mg/kg q 48 h	100	100	100	100	78	3	0	0	0	2.3	69.8	100
8 mg/kg	5 mg/kg q 48 h	100	100	100	100	96	17	0	0	0	13.2	72.2	100
Creatinine clearance 26–50 mL/min													
5 mg/kg	3 mg/kg q 24 h	100	100	100	100	78	3	0	0	0	1.9	69.8	100
5 mg/kg	4 mg/kg q 24 h	100	100	100	100	92	8	0	0	0	6.0	70.6	100
7 mg/kg	3 mg/kg q 24 h	100	100	100	100	79	3	0	0	0	2.3	69.8	100
7 mg/kg	4 mg/kg q 24 h	100	100	100	100	91	8	0	0	0	6.5	70.7	100
8 mg/kg	3 mg/kg q 24 h	100	100	100	100	79	3	0	0	0	2.3	69.8	100
8 mg/kg	4 mg/kg q 24 h	100	100	100	100	92	9	0	0	0	6.6	70.8	100
Creatinine clearance 51–90 mL/min													
7 mg/kg	5 mg/kg q 24 h	100	100	100	100	92	6	0	0	0	3.1	70.4	100
7 mg/kg	6 mg/kg q 24 h	100	100	100	100	96	12	0	0	0	5.8	71.3	100
8 mg/kg	5 mg/kg q 24 h	100	100	100	100	92	6	0	0	0	3.6	70.4	100
8 mg/kg	6 mg/kg q 24 h	100	100	100	100	96	12	0	0	0	6.4	71.3	100
8 mg/kg	7 mg/kg q 24 h	100	100	100	100	98	19	0	0	0	10.6	72.6	100
Creatinine clearance 91–130 mL/min													
7 mg/kg	5 mg/kg q 24 h	100	100	100	100	89	5	0	0	0	3.2	70	100
7 mg/kg	6 mg/kg q 24 h	100	100	100	100	94	11	0	0	0	6.8	71	100
8 mg/kg	5 mg/kg q 24 h	100	100	100	100	88	6	0	0	0	3.4	70	100
8 mg/kg	6 mg/kg q 24 h	100	100	100	100	94	11	0	0	0	6.2	71	100
8 mg/kg	7 mg/kg q 24 h	100	100	100	100	97	18	0	0	0	9.7	72	100

Abbreviations: AUC, area under the curve; MIC, minimum inhibitory concentration; LD, loading dose; MD, maintenance dose; TGC, tigecycline; GEN, gentamicin.

## Data Availability

Data available on the request due to restrictions.

## Supplementary Materials

The following are available online at https://www.mdpi.com/2079-6382/10/6/736/s1, Supplementary Materials S1: Table S1: A set of parameters of tigecycline; Table S2: A set of parameters and an equation of amikacin; Table S3: A set of parameters and an equation of gentamicin; Table S4: Antibiotic dosing regimens for simulation; Supplementary Materials S2: MIC distribution of single and combined antibiotics.
