# Correction: Nulsopapon et al. The Synergistic Activity and Optimizing Doses of Tigecycline in Combination with Aminoglycosides against Clinical Carbapenem-Resistant *Klebsiella pneumoniae* Isolates. *Antibiotics* 2021, *10*, 736

**DOI:** 10.3390/antibiotics11060745

**Published:** 2022-05-31

**Authors:** Parnrada Nulsopapon, Worapong Nasomsong, Manat Pongchaidecha, Dhitiwat Changpradub, Piraporn Juntanawiwat, Wichai Santimaleeworagun

**Affiliations:** 1 Department of Pharmacy, Faculty of Pharmacy, Silpakorn University, Nakhon Pathom 73000, Thailand; aom.olivegreen1812@gmail.com (P.N.); pongchaidecha_m@su.ac.th (M.P.); 2 Pharmaceutical Initiative for Resistant Bacteria and Infectious Diseases Working Group [PIRBIG], Faculty of Pharmacy, Silpakorn University, Nakhon Pathom 73000, Thailand; 3 Department of Internal Medicine, Division of Infectious Diseases, Phramongkutklao Hospital and College of Medicine, Bangkok 10400, Thailand; nasomsong.w@gmail.com (W.N.); dhitiwat@yahoo.com (D.C.); 4 Department of Clinical Pathology, Division of Microbiology, Phramongkutklao Hospital, Bangkok 10400, Thailand; mamicroma@gmail.com

## Text Correction

There were errors made in the original article [1]. The authors made additional revisions according to reviewers’ comments. Some sentences were changed and further explained. 

Corrections have been made as follows:

1. *Abstract:* “The synergy of tigecycline combined with amikacin or gentamicin was 8.2%” has been corrected to: “Synergistic activity was observed in 8.2% of isolates for tigecycline combined with amikacin or gentamicin”.

2. *Introduction, paragraph 3:* “The dose of antibiotics may be divided into carbapenem-based regimens (when meropenem MICs ≤ 8 µg/mL), colistin-based regimens, and tigecycline-based regimens, whereas aminoglycosides may be often used as adjunctive antibiotics [14]” has been corrected to: “The antibiotic regimens may be divided into carbapenem-based regimens (when meropenem MICs ≤ 8 µg/mL), colistin-based regimens, and tigecycline-based regimens, whereas aminoglycosides may be often used as adjunctive antibiotics [14]”.

3. *Introduction, paragraph 4:* “Therefore, high doses of tigecycline and/or the combination of tigecycline with other active agents may increase the plasma concentrations, leading to adequate bactericidal activity against CRE isolates [2]” has been corrected to: “Therefore, high doses of tigecycline may increase the plasma concentrations, leading to adequate bactericidal activity against CRE isolates [2]”.

4. *Materials and Methods, Section 2.2.1:* “Furthermore, a purified single colony of isolated strains was picked up and emulsified to 0.9% in normal saline” has been corrected to: “Furthermore, a purified single colony of isolated strains was picked up and suspended to 0.9% in normal saline”.

5. *Materials and Methods, Section 2.2.2:* “The tigecycline-based combination regimens consisted of tigecycline-amikacin and tigecycline-gentamicin. The microbroth checkerboard technique was used to evaluate the synergistic activity of each combination. The range of a series of two-fold dilutions was 0.125–8 μg/mL for tigecycline, 0.0625–64 μg/mL for amikacin, and 0.0078–8 μg/mL for gentamicin” has been corrected to: “The synergistic effect of antibiotic combinations including tigecycline-amikacin and tigecycline-gentamicin was determined using the checkerboard technique. The studied antibiotic concentrations of a series of two-fold dilutions were 0.125–8 µg/mL for tigecycline, 0.0625–64 µg/mL for amikacin, and 0.0078–8 µg/mL for gentamicin”.

6. *Materials and Methods, Section 2.4, paragraph 1*: “A two-compartment model with the linear pharmacokinetic behavior of tigecycline, amikacin, and gentamicin was used to generate the relationship between drug concentration levels and time [32–34]” has been corrected to: “The relationship between drug concentration levels and time was generated using a two-compartment model for tigecycline and amikacin and a one-compartment model for gentamicin with the linear pharmacokinetic behavior [32–34]. For amikacin and gentamicin, creatinine clearance (CrCL) was included to simulate the plasma aminoglycoside concentration-time using the equations containing CrCL as covariate factor (Supplementary Materials S1: Tables S1–S3)”.

7. *Materials and Methods, Section 2.4, paragraph 3:* “Details of the antibiotic dosing regimen are shown in Supplementary Materials Table S4” has been corrected to: “Details of the antibiotic dosing regimen are shown in Supplementary Materials S1 (Table S4)”.

8. *Materials and Methods, Section 2.4, a new paragraph 5 is added:* “MCS is a mathematical technique to simulate 10,000 virtual patients based on mean pharmacokinetic parameters (drug clearance or volume of distribution) and their distribution (standard derivation) in the compartment models (one or two compartments) for computing the plasma concentration and time among individual virtual patients. The C_max_ and calculated AUC values from the trapezoidal rule among simulated patients and the probability of target attainment (PTA) of the target PK/PD index were determined”.

9. *Materials and Methods, Section 2.4, new paragraph 6:* “The optimal dosing regimens defined as the dosing regimens reached greater than 90% of PTA for documented therapy and greater than 90% of CFR for empirical therapy” has been corrected to: “The optimal dosing regimens defined as the dosing regimens of each antibiotic MIC or the MIC values from the synergistic study reached greater than 90% of PTA for documented therapy and greater than 90% of CFR for empirical therapy”.

10. *Results, Section 3.2, paragraph 2*: The new sentence “Details of the MIC distribution of single or combined antibiotics are shown in Supplementary Materials S2.” has been added.

11. *Discussion, paragraph 3:* “An earlier study showed aminoglycoside dosage regimens (ranged from 5 to 30 mg/kg) achieved the PTA target at MICs of 0.5 μg/mL with the corresponding target of C_max_/MIC ≥ 10 [45]” has been corrected to: “An earlier study showed aminoglycoside dosage regimens (ranged from 5 to 30 mg/kg) achieved the PTA target at MICs of 0.5 µg/mL with the corresponding target of Cmax/MIC ≥ 10 [33]”.

12. *Discussion, paragraph 4:* “In prior studies, colistin resistance rates of CRKP isolates were only 25% [39], whereas colistin resistance rates of *K. pneumoniae* isolates ranged from 17.3% to 19% [18,46,47]” has been corrected to: “In prior studies, colistin resistance rates of CRKP isolates were only 25% [39], whereas colistin resistance rates of *K. pneumoniae* isolates ranged from 17.3% to 76.1% [18,45–47]”.

13. *Discussion, paragraph 6:* “Previous studies also reported tigecycline plus amikacin or gentamicin was effective against *K. pneumoniae* isolates. For tigecycline plus amikacin, one study showed synergism was 36.4%, whereas another study found synergism was 70% [19,50]” has been corrected to: “Related studies also reported tigecycline plus amikacin or gentamicin was effective against *K. pneumoniae* isolates. For tigecycline plus amikacin, one study showed the synergistic effect was 36.4%, whereas another study found the synergistic effect was 70% [19,50]”.

14. *Discussion, paragraph 8*: “After that, mistranslation of amino acids occurs, leading to damage to the bacterial cytoplasmic membrane and interfering with protein synthesis. These protein errors also lead to the reduction in β-lactamase expression. Therefore, both antibiotics likely enhance their target sites, contributing to synergistic effects [52,53]” has been corrected to: “Aminoglycosides lead to mistranslated amino acids, in turn leading to damage to the bacterial cytoplasmic membrane and interfering with protein synthesis; these protein errors also lead to reduced β-lactamase expression from aminoglycoside activities. Therefore, aminoglycosides likely enhance combined antibiotics to the target sites, contributing to synergistic effects [52,53]”.

15. *Supplementary Materials*: Supplementary Materials S2 were omitted from the previous version. This section has been corrected to: “The following are available online at https://www.mdpi.com/article/10.3390/antibiotics10060736/s1, Supplementary Materials S1: Table S1: A set of parameters of tigecycline; Table S2: A set of parameters and an equation of amikacin; Table S3: A set of parameters and an equation of gentamicin; Table S4: Antibiotic dosing regimens for simulation; Supplementary Materials S2: MIC distribution of single and combined antibiotics”.

16. *References:* Reference 45 was incorrect. It has been replaced with: “45. Eiamphungporn, W.; Yainoy, S.; Jumderm, C.; Tan-Arsuwongkul, R.; Tiengrim, S.; Thamlikitkul, V. Prevalence of the colistin resistance gene mcr-1 in colistin-resistant *Escherichia coli* and *Klebsiella pneumoniae* isolated from humans in Thailand. *J. Global Antimicrob. Resist.* **2018**, *15*, 32–35”.

The authors apologize for any inconvenience caused and state that the scientific conclusions are unaffected. The original article has been updated.

## References

[1] Nulsopapon P., Nasomsong W., Pongchaidecha M., Changpradub D., Juntanawiwat P., Santimaleeworagun W. (2021). The Synergistic Activity and Optimizing Doses of Tigecycline in Combination with Aminoglycosides against Clinical Carbapenem-Resistant Klebsiella pneumoniae Isolates. Antibiotics.

