# Supplementary material for: The Synergistic Activity and Optimizing Doses of Tigecycline in Combination with Aminoglycosides against Clinical Carbapenem-Resistant Klebsiella pneumoniae Isolates
Source: Antibiotics (Basel). 2021 Jun 17;10(6):736. doi: 10.3390/antibiotics10060736 (PMC8234075; doi:10.3390/antibiotics10060736)
Supplement: Supplementary file 1 [file antibiotics-10-00736-s001.zip › antibiotics-1219549-Supplementary Materials-final.pdf]

Supplementary Materials

Supplementary Material S1

Table S1 A set of parameters of tigecycline [34]

PK parameters	Estimate	% RSE
CL (L/h)	22.1	3.16
V_C (L)	162.0	5.3
Q (L/h)	69.4	32.6
V_P (L)	87.9	8.67

Abbreviations: CL, Clearance; V_C, Central volume of distribution; Q, intercompartmental clearance; V_P, Peripheral volume of distribution; RSE, Relative standard error

Table S2 A set of parameters and an equation of amikacin [32]

PK parameters	Estimates	% RSE
CL (L/h)	0.77	28.4
V_c (L)	19.2	5.31
Q (L/h)	4.38	18.3
V_P (L)	9.38	7.15
$\theta_{CL\text{-}CrCL}$ (ml/min)	1.42	18.4

Abbreviations: CL, Clearance; V_C, Central volume of distribution; Q, intercompartmental clearance; V_P, Peripheral volume of distribution; RSE, Relative standard error; $\theta_{CL\text{-}CrCL}$, fractional change on CL resulting from CrCL; CrCL, creatinine clearance (estimated by the Cockcroft-Gault)

Covariate model: $CL_{pop} = CL + CrCL^{\theta_{CL\text{-}CrCL}}$

Table S3 A set of parameters and an equation of gentamicin [33]

PK parameters	Estimate	% CV
CL (L/h)	3.14	83.7
V(L)	53.0	64.4

Abbreviations: CL, Clearance; V_C, Central volume of distribution; Q, intercompartmental clearance; V_P, Peripheral volume of distribution; CV, Coefficient of variation;

Covariate model: CL = $\frac{3.14\ x\ CrCL^{1.2}}{54.8^{1.2} + CrCL^{1.2}}$; CrCL, creatinine clearance (estimated by glomerular filtration rate (GFR): GFR (ml/min)= 186.3 X $Cr^{-1.154}$ X $Age^{-0.203}$ x (1.212 if black)x (0.742 if female)
where Cr is serum creatinine level (mg/dl)

Table S4 Antibiotic dosing regimens

Antibiotics	CrCL (ml/min)	LD	MD
Tigecycline	-	200 mg	100 mg q 12 h
		200 mg	100 mg q 24 h
		400 mg	100 mg q 12 h
		400 mg	100 mg q 24 h
		400 mg	200 mg q 12 h
		400 mg	200 mg q 24 h
Amikacin	0-9	15 mg/kg	7.5 mg/kg q 48 h
		20 mg/kg	7.5 mg/kg q 48 h
		25 mg/kg	7.5 mg/kg q 48 h
	10-25	20 mg/kg	10 mg/kg q 48 h
		20 mg/kg	15 mg/kg q 48 h
		25 mg/kg	10 mg/kg q 48 h
		25 mg/kg	15 mg/kg q 48 h
	26-50	20 mg/kg	12 mg/kg q 24 h
		20 mg/kg	15 mg/kg q 24 h
		25 mg/kg	12 mg/kg q 24 h
		25 mg/kg	15 mg/kg q 24 h
	51-90	25 mg/kg	15 mg/kg q 24 h
		25 mg/kg	20 mg/kg q 24 h
		30 mg/kg	15 mg/kg q 24 h
		30 mg/kg	20 mg/kg q 24 h
	91-130	25 mg/kg	15 mg/kg q 24 h
		25 mg/kg	20 mg/kg q 24 h
		30 mg/kg	15 mg/kg q 24 h
		30 mg/kg	20 mg/kg q 24 h

Antibiotics	CrCL (ml/min)	LD	MD
Gentamicin	0-9	3 mg/kg	2.5 mg/kg q 48 h
		5 mg/kg	2.5 mg/kg q 48 h
		7 mg/kg	2.5 mg/kg q 48 h
	10-25	5 mg/kg	4 mg/kg q 48 h
		7 mg/kg	3 mg/kg q 48 h
		7 mg/kg	5 mg/kg q 48 h
		8 mg/kg	3 mg/kg q 48 h
		8 mg/kg	5 mg/kg q 48 h
	26-50	5 mg/kg	3 mg/kg q 24 h
		5 mg/kg	4 mg/kg q 24 h
		7 mg/kg	3 mg/kg q 24 h
		8 mg/kg	7 mg/kg q 24 h
	51-90	7 mg/kg	5 mg/kg q 24 h
		7 mg/kg	6 mg/kg q 24 h
		8 mg/kg	5 mg/kg q 24 h
		8 mg/kg	6 mg/kg q 24 h
		8 mg/kg	7 mg/kg q 24 h
	91-130	7 mg/kg	5 mg/kg q 24 h
		7 mg/kg	6 mg/kg q 24 h
		8 mg/kg	5 mg/kg q 24 h
		8 mg/kg	6 mg/kg q 24 h
		8 mg/kg	7 mg/kg q 24 h

Abbreviations: CrCL, Creatinine clearance; LD, Loading dose; MD, Maintenance dose

Supplementary Material S2

Table S5: Tigecycline MIC distribution (monotherapy)

Tigecycline MIC (monotherapy) (μg/ml)	Frequency (n)	Percentage (%)
≤ 0.03125	0	0.00
0.0625	0	0.00
0.125	0	0.00
0.25	0	0.00
0.5	10	20.41
1	18	36.73
2	18	36.73
4	1	2.04
8	1	2.04
16	1	2.04
32	0	0.00
64	0	0.00
128	0	0.00

Table S6: Amikacin MIC distribution (monotherapy)

Amikacin MIC (monotherapy) (μg/ml)	Frequency (n)	Percentage (%)
≤ 0.0625	0	0.00
0.125	0	0.00
0.25	1	2.04
0.5	1	2.04
1	3	6.12
2	16	32.65
4	6	12.24
8	14	28.57
16	4	8.16
32	4	8.16
64	0	0.00
128	0	0.00

Table S7: Amikacin MIC distribution (combination therapy)

Amikacin MIC (combined with tigecycline) (μg/ml)	Frequency (n)	Percentage (%)
≤ 0.0625	17	34.69
0.125	10	20.41
0.25	9	18.37
0.5	6	12.24
1	1	2.04
2	6	12.24
4	0	0.00
8	0	0.00
16	0	0.00
32	0	0.00
64	0	0.00

Table S8: Gentamicin MIC distribution (monotherapy)

Gentamicin MIC (monotherapy) (μg/ml)	Frequency (n)	Percentage (%)
≤ 0.03125	9	18.37
0.0625	2	4.08
0.125	11	22.45
0.25	8	16.33
0.5	4	8.16
1	0	0.00
2	8	16.33
4	2	4.08
8	3	6.12
16	1	2.04
32	0	0.00
64	1	2.04

Table S9: Gentamicin MIC distribution (combination therapy)

Gentamicin MIC (combined with tigecycline) (μg/ml)	Frequency (n)	Percentage (%)
≤ 0.0078125	26	53.06
0.015625	2	4.08
0.03125	8	16.33
0.0625	6	12.24
0.125	7	14.29
0.25	0	0.00
0.5	0	0.00
1	0	0.00
2	0	0.00
4	0	0.00
8	0	0.00
